# Atrial Bradycardia

**Published:** 1969-10

**Authors:** David B. Shaw, Dennis Eraut


### ATRIAL BRADYCARDIA OR THE LAZY SINUS SYNDROME

**David B. Shaw and Dennis Eraut**

This paper describes a group of patients who have chronic bradycardia, due to a slow atrial rate. This can be the result of sinus bradycardia or produced by block between the sinus node and the atrium. The distinction between the two processes may be difficult, since electrical discharge of the sinus node does not give rise to a deflection on the standard electrocardiogram. For a start, therefore, they will be grouped together as atrial bradycardia.

There are three common groups of causes for atrial bradycardia: physiological, in association with an increased vagal tone in normal subjects; drug induced, as with digoxin, propranolol and the newer hypotensive agents; and in association with certain disease states, such as myxœdema, hypopituitarism, and raised intra-cranial tension. However, five years ago we saw three patients with atrial rates of about 40 per minute which could not be explained by any of these processes, and we therefore started to collect patients with the condition.

The initial criterion for admission to the study was a resting atrial rate of below 56 per minute. Patients on drugs known to slow the heart were excluded, as were those with systemic diseases normally associated with bradycardia, such as myxœdema. The study was primarily for chronic bradycardia and patients with temporary slowing of the heart during acute cardiac infarction or acute carditis were not included.

Thirty-eight patients were found who complied with the criteria of the study. Of these, thirteen were seen in routine hospital practice, while an additional twenty-five were picked up in the Devon Heart-Block Survey. This survey covered 290 family doctors in the Exeter area, who between them looked after a population of approximately 600,000 people. The family doctors were sent a circular asking for details of patients with known disturbance of atrio-ventricular conduction, or with pulse rates below 56 per minute. The doctors were in fact very co-operative and we had replies from 282.

The patients were seen either in the Cardiac Department at the Royal Devon and Exeter Hospital, or at their homes. A full clinical history was obtained and a physical examination was performed. A 12-lead electrocardiogram was then recorded and the resting heart rate was taken from a 3 foot strip of lead 2 of the cardiogram. The average rate for the group of 38 patients was 44 beats per minute. The distribution of rates are given in Table I; at the top end of the scale, 2 patients had resting rates of 55 per minute, while at the other end 1 had a rate below 30 per minute. Approximately half of the patients had rates below 45 per minute.

**TABLE I. Mean resting Heart Rates in 38 patients.**

| | Heart rate per minute | | | | | | | |
|---|---|---|---|---|---|---|---|---|
| | **20-24** | **25-29** | **30-34** | **35-39** | **40-44** | **45-49** | **50-54** | **55** |
| No. of Patients | 1 | 0 | 4 | 3 | 9 | 6 | 13 | 2 |

The age range was very wide, being from 13 to 83. However, most of the patients tended to be elderly and more than half lay within the age range 60

to 80 years. 30 of the 38 patients had symptoms suggestive of circulatory impairment. 16 experienced some transitory disturbance of consciousness. In 11 of these there was complete loss of consciousness, while 9 had attacks of faintness or dizziness. 4 patients experienced both types of disturbance. Angina of effort was present in 10 subjects. This seemed a little surprising in view of the current therapeutic fashion of treating angina with propranolol, which induces sinus bradycardia. Breathlessness on exercise was a common symptom, being present in 23 patients; 2 patients had ankle œdema, secondary to congestive cardiac failure.

Some of the symptoms complained of by these patients might theoretically be due to associated or coincidental cardiac disease, rather than to the bradycardia. However, in practice the evidence of such disease was rare. The commonest associated condition was that of coronary artery disease which was present in 8 of the 38 patients. This heading included those who gave a past history of myocardial infarction and all patients with cardiographic changes of infarction or ischæmia (groups 1AB and group 2 in the World Health Organisation criteria for cardiac infarction). Of the 10 patients with symptoms of angina of effort, 6 came within this group. 2 patients had evidence of rheumatic valvular disease, 1 had congenital heart disease and 1 was presumed to have a fibrotic type of cardiomyopathy. 26 of the subjects had no evidence of cardiac disease other than the slow heart rate.

The criterion for selection was such that the initial cardiogram in all the patients showed atrial bradycardia. However, at the time of their most recent assessment, the atrio-ventricular node had become the dominant pacemaker in 4 subjects, while in 6, atrial fibrillation or flutter had replaced the atrial bradycardia (Table II). An additional patient had developed atrial fibrillation, but successfully reverted to sinus rhythm with D.C. shock.

**TABLE II. Predominant Rhythm at MOST RECENT assessment.**

| | |
|---|---|
| Atrial bradycardia ... ... | 28 |
| Nodal rhythm ... ... ... | 4 |
| Atrial flutter or fibrillation ... | 6 |
| TOTAL | 38 |

When present, the P-waves of the cardiograph were of low voltage; they were often difficult to distinguish at all in standard lead 1, in lead 2 they were often broad and bifid. Generally, the P-P intervals were long and relatively constant. However, periodically, the atrium did not depolarize on time, and a nodal escape-beat occurred. These pauses in P-wave production were not related to respiration, nor did the P-P intervals commonly show sinus arrhythmia. It would seem, therefore, that either the sinus node failed to fire on these occasions, or that the impulse was blocked between the sinus node and the atrium. The two mechanisms can often be distinguished by their association either with nodal escape-beats or with sudden doubling of the atrial rate. Escape-beats are said to be rare in sino-atrial block, but common in sinus bradycardia (Stock 1969). Sudden doubling of the atrial rate can be expected in sino-atrial block when the degree of block changes. This may occur at rest or in response to exercise or drugs. In sinus bradycardia no such change in atrial rate would be expected.

Escape-beats proved to be common in the patients of this study, while doubling of the heart rate was only recorded in 1 of the 38 patients, either at rest or with exercise. The typical response to exercise was a trivial increase in *atrial* rate and a rate above 60 per minute was only recorded in 6 patients. However, occasionally, a larger increase in *ventricular* rate occurred when the atrio-ventricular node took over as pacemaker. Atropine and isoprenaline produced similar effects on heart rate to those of exercise in the 4 patients to whom these drugs were administered.

On the evidence presented it seemed unlikely that sino-atrial block could have accounted for the bradycardia in the majority of patients. Rather, it is likely that the inherent rhythmicity of the sino-atrial node was at fault. This leads us to refer to the condition as the lazy sinus syndrome. The ætiology remains a mystery. It has been suggested that certain cerebral lesions can produce sinus bradycardia, but evidence of neurological disease was found in only 6 patients. 3 of those had had cerebro-vascular accidents, 1 had evidence of cerebral degeneration and 2 were at one time considered to have epilepsy.

In summary, 38 patients were encountered who had a marked bradycardia but no significant interference with atrio-ventricular conduction. The majority had symptoms of cardiovascular disease including a third with syncopal attacks and a quarter with angina of effort. Nevertheless, few had evidence of heart disease other than the bradycardia. It was likely that one or two subjects were included who had simple physiological bradycardia. However, sino-atrial block was confirmed in only 1 patient, and for the reasons presented it was suspected that the majority had sinus bradycardia. It is suggested that the primary pathology lies in the sino-atrial node and it is likely to be significant that 7 of the 38 patients in the study developed atrial fibrillation (a condition in which Hudson (1960) reports degenerative changes of the sinus node). While awaiting further enlightenment of the cause of the sino-atrial node, we have christened this the lazy sinus syndrome.

*Acknowledgements*

We would like to acknowledge the help and co-operation of our colleagues in general practice and in the hospital service, in telling us about their patients. Part of this work was financed by grants from the Ministry of Health and from the South Western Regional Hospital Board.

### REFERENCES

Hudson, R. E. B. (1960), The Human Pacemaker and its Pathology. Brit. Heart J. *22*, 153.

Stock, J. P. P. (1969), Diagnosis and Treatment of Cardiac Arrhythmias. Butterworths, LONDON.

